# Role of HIF-1a in regulating autophagic cell survival during cerebral ischemia reperfusion in rats

**DOI:** 10.18632/oncotarget.21445

**Published:** 2017-10-01

**Authors:** Yongqing Guo

**Affiliations:** ^1^ Department of Anesthesiology, Shanxi Provincial People's Hospital, Taiyuan 030012, China

**Keywords:** autophagy, apoptosis, cerebral ischemia reperfusion, HIF-1a

## Abstract

Hypoxia-inducible factor-1a (HIF-1a) plays a beneficial role during cerebral ischemia reperfusion (IR), but the underlying molecular mechanisms are not completely understood. Here, we aimed to investigate the effects and molecular regulation of HIF-1a on brain cell apoptosis and autophagy during IR. We found that augmentation of HIF-1a in re-perfused hematopoietic cells significantly reduced brain damage, alleviated brain edema and improved neural function during IR, seemingly through two HIF-1a target genes BNIP3 and NIX, which were critical regulators for cell apoptosis and autophagic cell survival. *in vitro*, HIF-1a induced up-regulation of BNIP3 and NIX in human cortical neuron cells, HCN-1A. Inhibition of BNIP3 and NIX significantly attenuated HIF-1a-suppressed cell apoptosis and HIF-1a-induced cell autophagy. Together, these data suggest that HIF-1a may ameliorate brain damages during IR through BNIP3 and NIX -dependent augmentation of autophagic cell survival and reduction in cell apoptosis.

## INTRODUCTION

The human brain is one of the most vulnerable and sensitive organs to ischemia due to basic anaerobic metabolism and low glycogen stores. Many diseases may cause temporary global cerebral ischemia, e.g. cardiac arrest and shock. Among all regions in the brain, the hippocampal CA1 region appears to be affected more significantly by ischemia [1].

Cerebral ischemia reperfusion (IR) causes cerebral injury and brain dysfunction due to oxidative damage and apoptotic cell death [2, 3]. Although it is critical to provide reperfusion to the ischemic tissue at earliest stage, the outcome may not be ideal due to the presence of free oxygen radicals in IR [4]. Delayed neural cell death during IR injuries mainly result from cell apoptosis [4]. It has been shown that brain could be protected via the reduction in inflammation and apoptosis after IR [5].

Hypoxia-inducible factor 1a (HIF-1a) is the regulatory subunit of a master regulator of hypoxia –HIF-1 [6–8]. HIF-1a regulates the expression of genes encoding molecules that participate in erythropoiesis, cell proliferation, and energy metabolism, and is closely associated with the regulation of neuronal survival in ischemia [6–8]. However, the effects of HIF-1a on cell death or survival is complicated and may be dependent on the levels of HIF-1a and on the cell type and situation [6–8]. Caspases play an essential role in the development of apoptosis. Interestingly, HIF-1a has been shown to bind functionally to the caspase 3 gene promoter [9]. However, the neuroprotective mechanism of HIF-1a in the setting of IR, especially through regulation of autophagy, is not elucidated.

Autophagy was initially characterized based on its ultrastructural features, in particular, double-membraned structures that surrounded cytoplasm and organelles in cells, known as autophagosomes [10–13]. Autophagy is a catabolic program, which is activated in response to starvation or alteration in nutrient conditions [10–13]. Previous studies have identified a number of proteins, and elucidated several biochemical pathways, that are essential for autophagy. Microtubule-associated protein 1A/1B-light chain 3 (LC3) is a soluble cellular protein and a marker for autophagy. During autophagy, autophagosomes engulf cytoplasmic components, resulting in changes of the cytosolic LC3-I into LC3-II. Thus, the ratio of LC3-II to LC3-I has been widely used as a marker of the autophagic activity [10–13]. Among all these autophagy-associated proteins, autophagy-related protein 6 (ATG6, or Beclin-1) appears to be a critical one, which is a component of a class III PI3-K-containing complex [14]. Recent studies have demonstrated a critical role of autophagy in the brain cell survival during IR [3, 15–21]. Nevertheless, regulation of brain cell autophagy by HIF-1a in IR has not been extensively reported.

BNIP3 (BCL2 and adenovirus E1B 19-kDa-interacting protein 3) and BNIP3-like (BNIP3L), also known as NIX, are proteins with homology to BCL2 in the BH3 domain, are both direct target genes of HIF-1a [22]. BNIP3 and NIX were initially regarded as regulators for apoptotic cell death, and recently discovered to both regulate autophagic cell survival as well [23, 24]. For example, in muscle wasting disorders, where autophagy is implicated in the pathogenesis, BNIP3 and NIX are shown to upregulate in skeletal muscle to induce autophagosome formation [25]. Thus, it is clear that BNIP3 and NIX exhibit a dual nature, in which they control both apoptosis and autophagy. However, a necessary role of BNIP3 and NIX in HIF-1a-mediated regulation of brain cell death or survival in IR has not been acknowledged.

Here, we aimed to investigate the effects and molecular regulation of HIF-1a on brain cell apoptosis and autophagy during IR. We used both an *in vivo* rat IR model and *in vitro* analyses of brain neural cells to study the effects of HIF-1a and its downstream signal cascades on brain cell apoptosis and autophagy. Depletion of BNIP3 and NIX *in vitro* was further used as loss-of-function *in vitro* to evaluate the necessity of them for HIF-1a-induced effects on brain cell apoptosis and autophagy.

## RESULTS

### Beneficial effects of HIF-1a during IR

**Figure 1:**
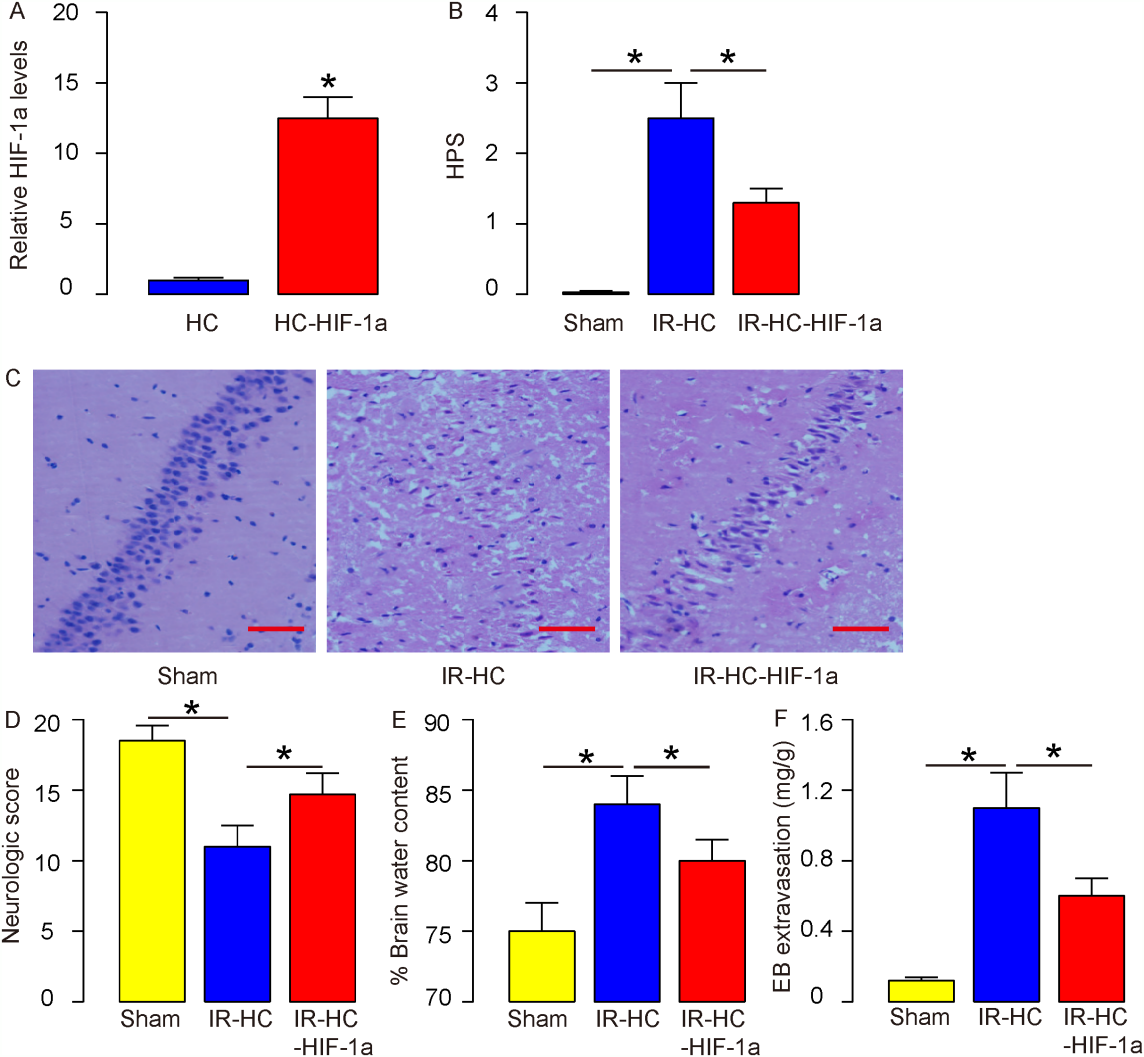
Beneficial effects of HIF-1a during IR **(A)** Rat hematopoietic cells (HCs) were transduced them with lentivirus carrying either null (as a control) or recombinant HIF-1a under a CMV promoter. HIF-1a levels in the transduced HCs were determined by HIF-1a ELISA. **(B-C)** For HC reinfusion, 10^7^ donor null/HIF-1a-transduced HCs were given to the receipt rats 3 days before IR. The rats were randomly divided into 3 groups of 20 each: sham-treated (no IR; Sham); IR in rats that received 10^7^ donor null-transduced HCs 3 days before IR (IR-HC); IR in rats that received 10^7^ donor HIF-1a-transduced HCs 3 days before IR (IR-HC-HIF-1a). Histopathologic score (HPS) was done, shown by quantification (B), and by representative images (C). **(D)** The neurologic scores in 3 groups. **(E)** The brain water content in 3 groups. **(F)** The Brain-Blood Barrier (BBB) permeability was evaluated, using EB extravasation. N=20. ^*^p<0.05. Scale bars are 50μm.

In order to evaluate the effects of HIF-1a on brain damages and neural function during IR, we isolated rat hematopoietic cells (HCs) and transduced them with lentivirus carrying either null (as a control) or recombinant HIF-1a under a CMV promoter. The increases in HIF-1a levels in the transduced HCs were confirmed by HIF-1a ELISA (Figure 1A). For HC reinfusion, 10^7^ donor null/HIF-1a-transduced HCs were given to the receipt rats 3 days before IR. The rats were randomly divided into 3 groups of 20 each: sham-treated (no IR; Sham); IR in rats that received 10^7^ donor null-transduced HCs 3 days before IR (IR-HC); IR in rats that received 10^7^ donor HIF-1a-transduced HCs 3 days before IR (IR-HC-HIF-1a).

At analysis, we evaluated the histopathologic score (HPS) and found that IR significantly increased HPS, which was significantly attenuated by reperfusion with HC-HIF-1a (Figures 1B-1C). Neurologic scores were then analyzed, and we found that neurologic scores in the IR+HC group and the IR+HC-HIF-1a group were both significantly lower than in the Sham group after IR. However, HC-HIF-1a significantly improved the neurologic scores compared with the IR+HC group (Figure 1D). We then analyzed the brain water content. When we found that the brain water content in the IR+HC group and the IR+HC-HIF-1a group were both significantly higher than in the Sham group after IR. However, HC-HIF-1a significantly reduced the brain water content compared with the IR+HC group (Figure 1E). The effect of IR and HIF-1a on Brain-Blood Barrier (BBB) permeability were then evaluated, using EB extravasation as a marker. We found that after IR, a significant extravasation of EB, which is an index of BBB disruption, was detected in the IR+HC group and the IR+HC-HIF-1a group, compared to the Sham group. HIF-1a significantly decreased EB extravasation by IR (Figure 1F). Together, these data suggest that while IR induces functional damages in brain, augmentation of HIF-1a may attenuate these injuries to the brain.

### Augmentation of HIF-1a attenuates rat brain cell apoptosis after IR

**Figure 2:**
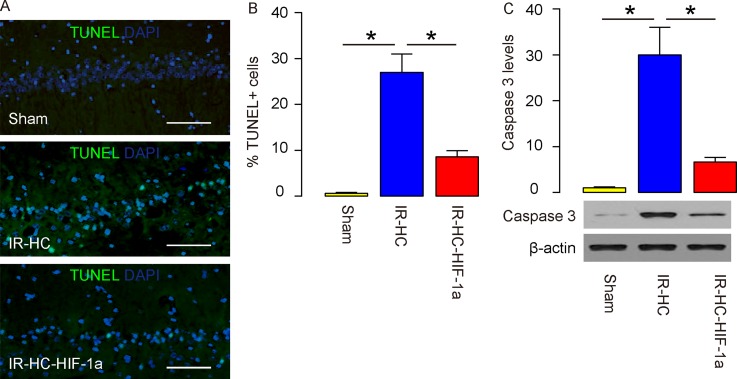
Augmentation of HIF-1a attenuates rat brain cell apoptosis after IR **(A-B)** TUNEL staining was then performed on the rat brain (Sham group, IR+HC group and IR+HC-HIF-1a group) after IR. The number of TUNEL-positive cells was shown by representative images (A), and by quantification (B). **(C)** Western blot for cleavage caspase 3 in brain. N=20. ^*^p<0.05. Scale bars are 50μm.

TUNEL staining was then performed on the rat brain at analysis. We found that while nearly no TUNEL-positive cells were found in the hippocampus in the Sham group, an increased number of TUNEL-positive cells were detected in the IR+HC group. HIF-1a significantly reduced the number of TUNEL-positive cells, shown by representative images (Figure 2A), and by quantification (Figure 2B). Moreover, a key protein for apoptosis, cleavage caspase 3, was significantly upregulated in the brain from IR+HC group and the IR+HC-HIF-1a group, compared to the Sham group. HIF-1a significantly decreased the levels of cleavage caspase 3 by IR, analyzed by Western blot (Figure 2C). Together, these data suggest that augmentation of HIF-1a may attenuate rat brain cellapoptosis after IR.

### HIF-1a enhances rat brain cell autophagy after IR

**Figure 3:**
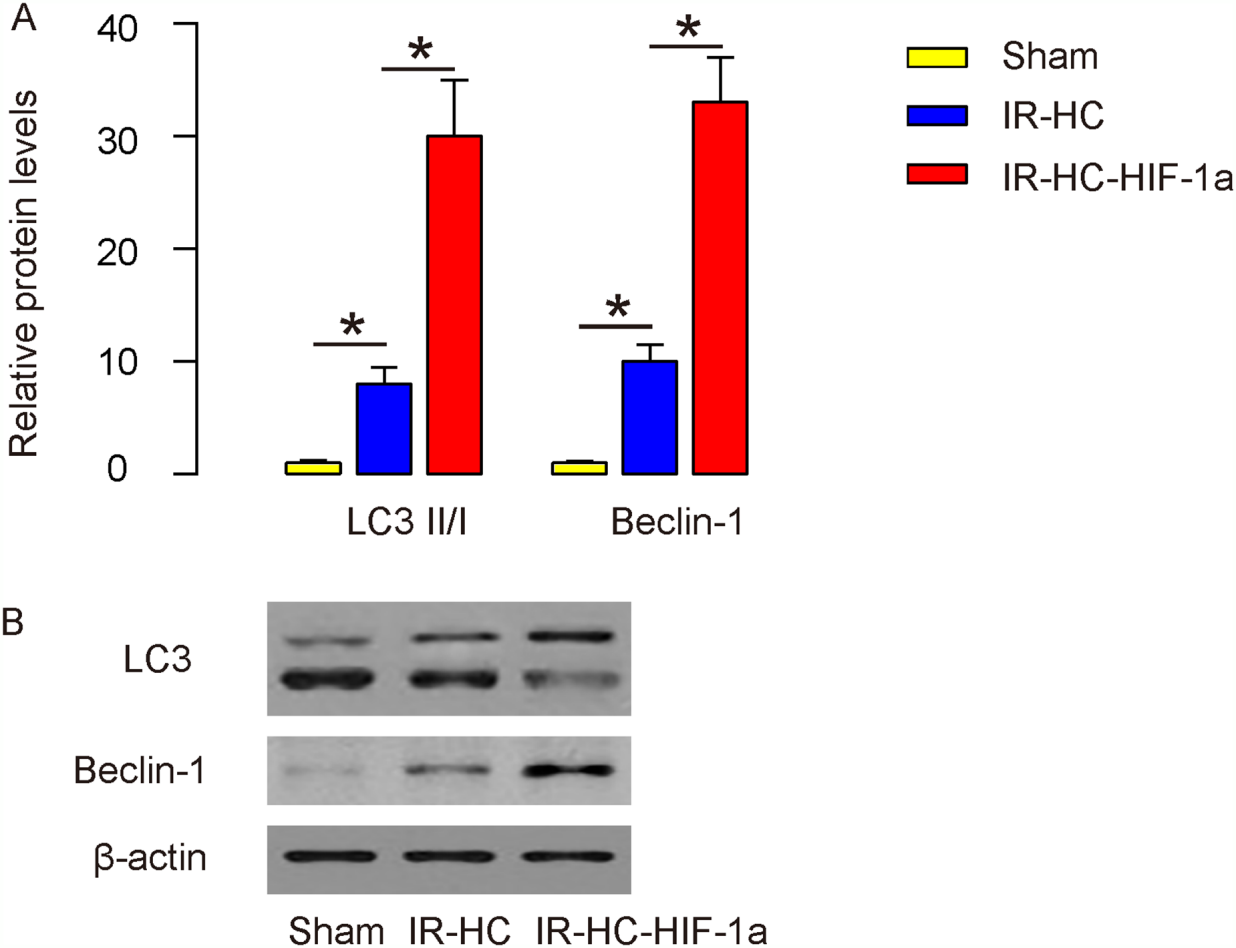
HIF-1a enhances rat brain cell autophagy after IR **(A-B)** Western blot for LC3 and Beclin-1 for rat brain tissue (Sham group, IR+HC group and IR+HC-HIF-1a group) after IR, shown by quantification (A), and by representative Western blots (B). LC3II/I: the ratio of LC3 II vs LC3 I. N=20. ^*^p<0.05.

Next, we analyzed the effects of IR and HIF-1a on rat brain cell autophagy after IR. The ratio of LC3 II to LC3 I is a marker for autophagy. We found that the LC3 II/I increased in rat brain after IR, and further significantly increased in rat brain by HIF-1a, shown by quantification (Figure 3A), and by representative Western blots (Figure 3B). These data suggest that while IR induced brain cell autophagy, which was further enhanced by HIF-1a by IR. Since Beclin-1 is a key autophagy-associated protein, we then analyzed the levels of Beclin-1. We found that the Beclin-1 levels significantly increased after IR, and further significantly increased in rat brain by HIF-1a, shown by quantification (Figure 3A), and by representative Western blots (Figure 3B), consistent with our findings in LC3. Thus, HIF-1a may enhance rat brain cell autophagy in after IR.

### IR induces HIF-1a and its two target genes BNIP3 and NIX in rat brain

**Figure 4:**
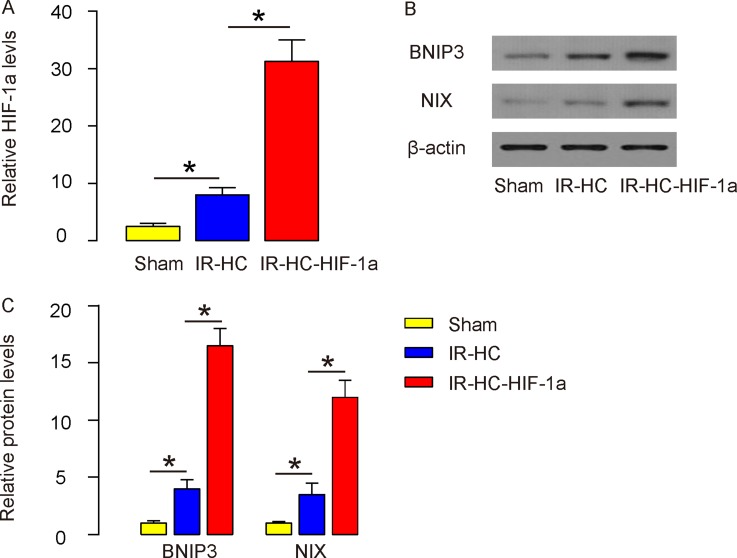
IR induces HIF-1a and its two target genes BNIP3 and NIX in rat brain **(A-C)** The levels of HIF-1a (by ELISA, A), BNIP3 and NIX (by Western blot, B-C) were examined in rat brain tissue (Sham group, IR+HC group and IR+HC-HIF-1a group) after IR. N=20. ^*^p<0.05.

Next, we examined the levels of HIF-1a in rat brain after IR. We found that HIF-1a was significantly upregulated in the brain from IR+HC group and the IR+HC-HIF-1a group, compared to the Sham group. Presence of HC-HIF-1a further increased the levels of HIF-1a in rat brain, analyzed by ELISA (Figure 4A). Then we screened the HIF-1a downstream target genes, and we specifically found that BNIP3 and NIX levels were similarly altered as HIF-1a (Figures 4A-4B).

### BNIP3 and NIX levels were similarly altered by HIF-1a *in vitro*

Then we used *in vitro* experiment to study the underlying mechanisms. Ischemic-like conditions for IR were mimicked by using oxygen-glucose deprivation and reperfusion (OGDR) *in vitro*. A human cortical neuron cell line, HCN-1A, was used. Cells that were not exposed to OGDR were used as controls. HIF-1a was induced in HCN-1A through transfection of cells with a HIF-1a-expressing plasmid under CMV promoter. HIF-1a was also transfected with a scrambled plasmid as a control. Depletion of the BNIP3 and NIX expression was achieved through transfection of the cells with shRNA for both (shBNIP3+shNIX). Four groups of the cells were compared: Group 1, scrambled plasmid transfected cells, no OGDR (Control); Group 2, scrambled plasmid transfected cells, OGDR (OGDR); Group 3, HIF-1a-transfected cells, OGDR (OGDR+HIF-1a); Group 4, HIF-1a/shBINP3/shNIX-transfected cells, OGDR (OGDR+HIF-1a+shBNIP3+shNIX).

**Figure 5:**
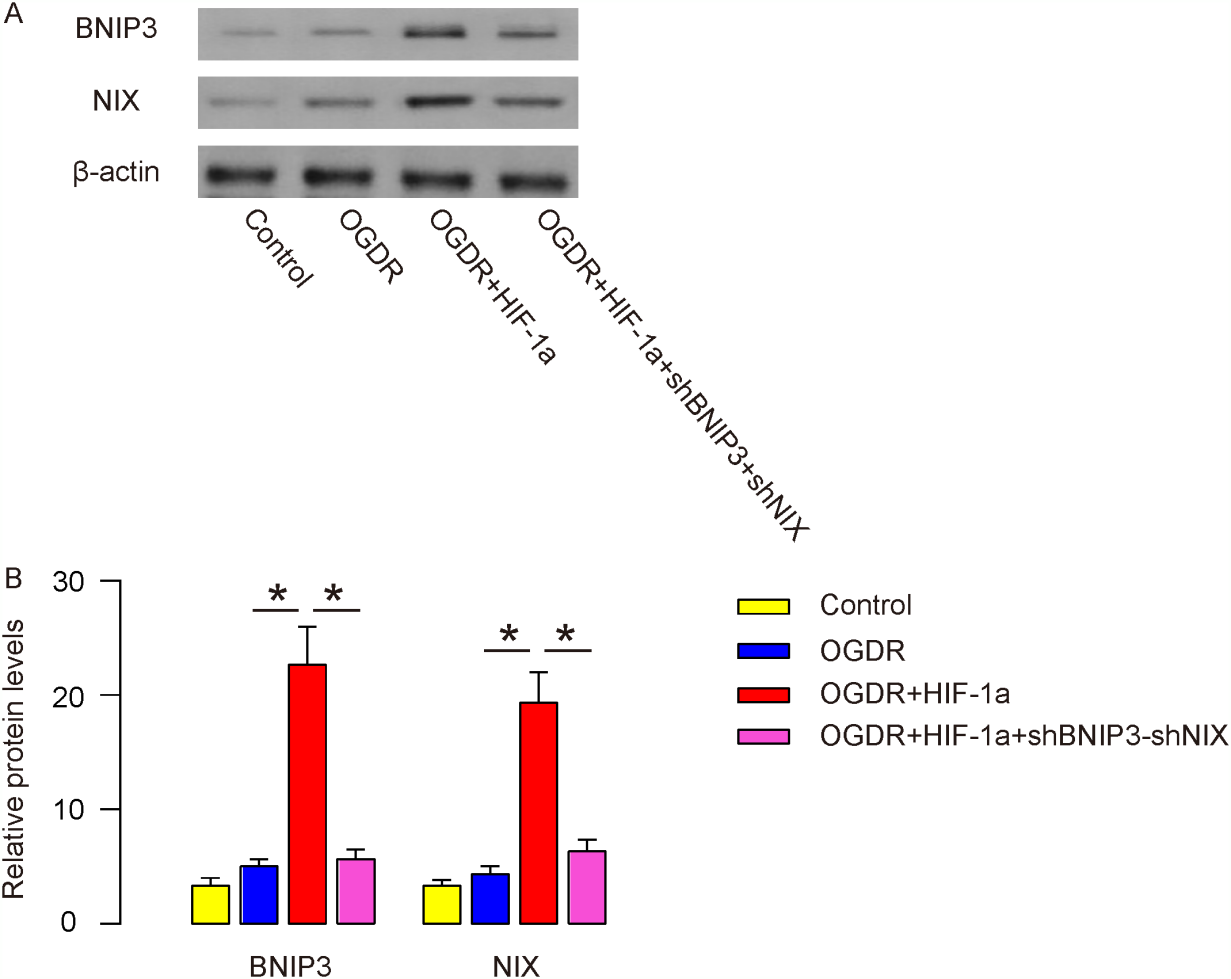
BNIP3 and NIX levels were similarly altered by HIF-1a *in vitro* Ischemic-like conditions for IR were mimicked by using oxygen-glucose deprivation and reperfusion (OGDR) *in vitro*. A human cortical neuron cell line, HCN-1A, was used. Cells that were not exposed to OGDR were used as controls. HIF-1a was induced in HCN-1A through transfection of cells with a HIF-1a-expressing plasmid under CMV promoter. HIF-1a was also transfected with a scrambled plasmid as a control. Depletion of the BNIP3 and NIX expression was achieved through transfection of the cells with shRNA for both (shBNIP3+shNIX). Four groups of the cells were compared: Group 1, scrambled plasmid transfected cells, no OGDR (Control); Group 2, scrambled plasmid transfected cells, OGDR (OGDR); Group 3, HIF-1a-transfected cells, OGDR (OGDR+HIF-1a); Group 4, HIF-1a/shBINP3/shNIX-transfected cells, OGDR (OGDR+HIF-1a+shBNIP3+shNIX). **(A-B)** Western blot for BNIP3 and NIX were examined in in HCN-1A cells, shown by representative Western blots (A), and by quantification. ^*^p<0.05. N=5.

We found that induction of HIF-1a in HCN-1A cells significantly increased BNIP3 and NIX, which was reduced by depletion of BNIP3 and NIX in cells, shown by representative Western blots (Figure 5A), and by quantification (Figure 5B). Thus, BNIP3 and NIX levels are similarly altered by HIF-1a *in vitro*.

### HIF-1a attenuates OGDR-induced brain cell apoptosis via BNIP3 and NIX *in vitro*

**Figure 6:**
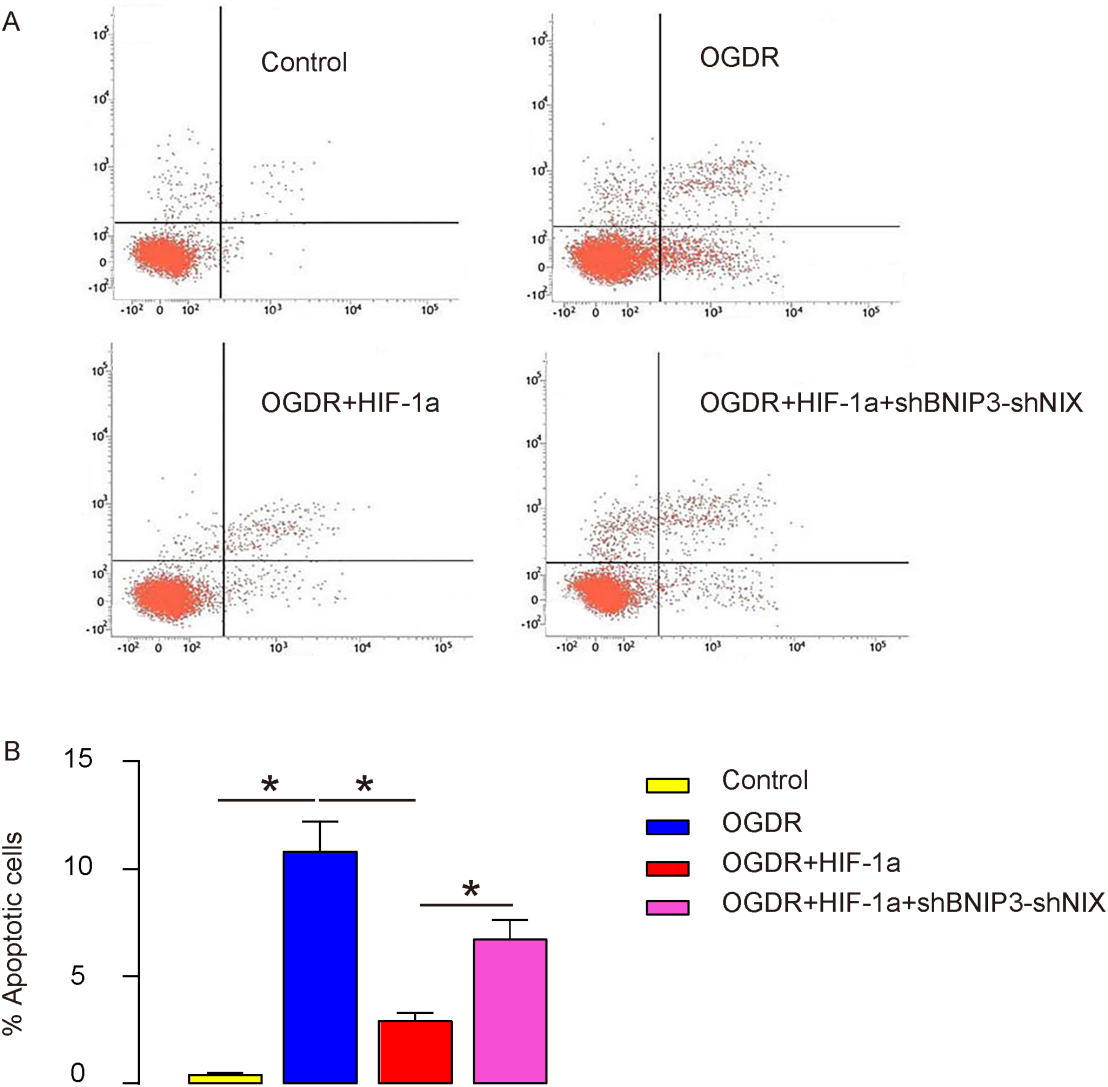
HIF-1a attenuates OGDR-induced brain cell apoptosis via BNIP3 and NIX *in vitro* **(A-B)** Cell apoptosis was analyzed by flow cytometry-based FITC Annexin V Apoptosis Detection Assay, shown by representative flow charts (A), and by quantification (B). ^*^p<0.05. N=5.

Cell apoptosis was then analyzed by flow cytometry-based FITC Annexin V Apoptosis Detection Assay. We found that while nearly no apoptotic cells were found in the Control group, an increased number of apoptotic cells were detected in OGDR group. Augmentation of HIF-1a significantly reduced the number of apoptotic cells, which was abolished by BNIP3 and NIX depletion, shown by representative flow charts (Figure 6A), and by quantification (Figure 6B). Thus, HIF-1a attenuates OGDR-induced brain cell apoptosis via BNIP3 and NIX *in vitro*.

### HIF-1a enhances OGDR-associated brain cell autophagy via BNIP3 and NIX *in vitro*

**Figure 7:**
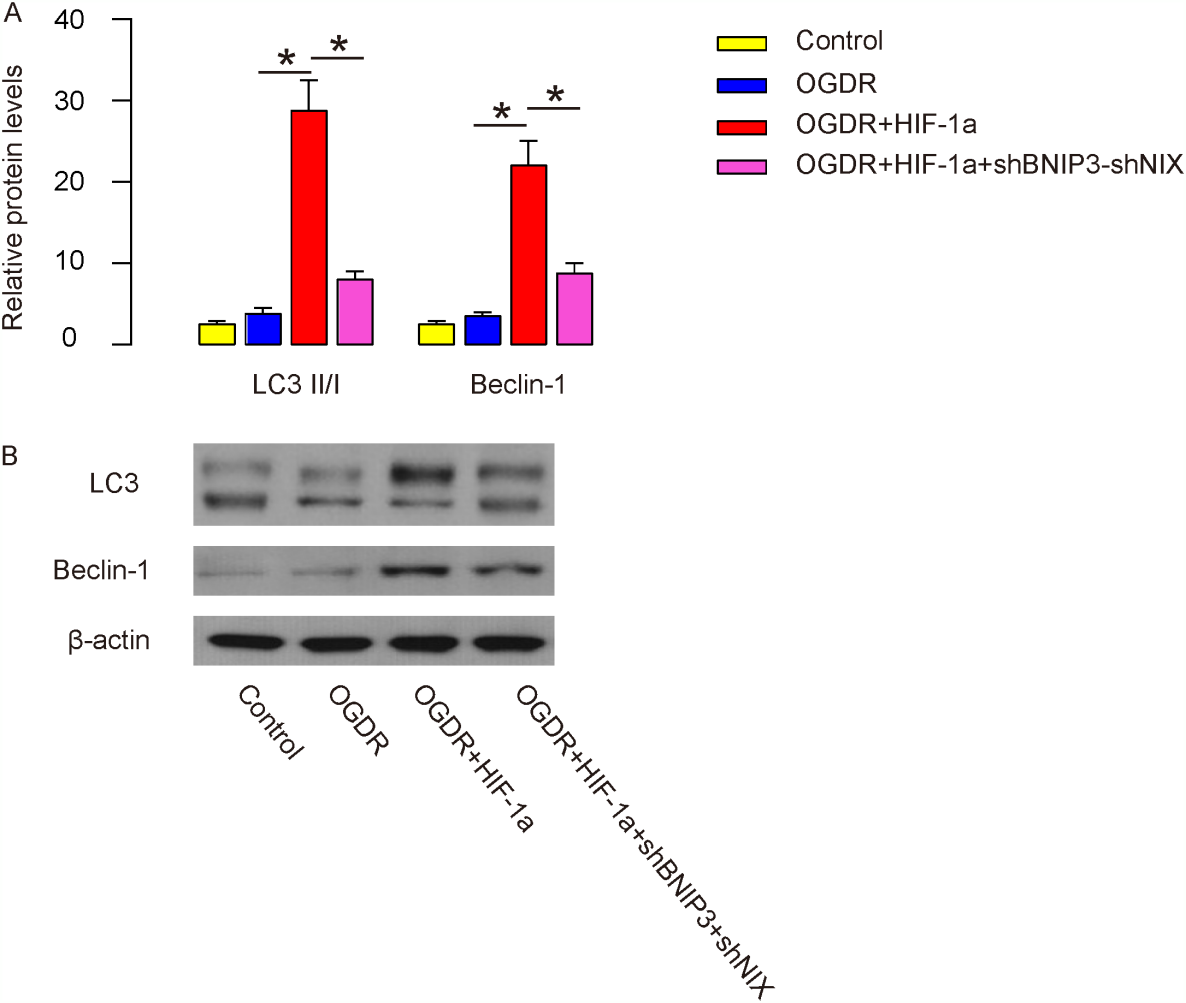
HIF-1a enhances OGDR-associated brain cell autophagy via BNIP3 and NIX *in vitro* **(A-B)** Cell autophagy was analyzed. LC3II/I and Beclin-1 were analyzed by Western blot, shown by quantification (A), and by representative Western blots (B). ^*^p<0.05. N=5.

Cell autophagy was then analyzed. We found that the levels of LC3II/I and Beclin-1 significantly increased by HIF-1a, which was abolished by BNIP3 and NIX depletion, shown by quantification (Figure 7A), and by representative Western blots (Figure 7B). Thus, HIF-1a enhances brain cell autophagy via BNIP3 and NIX *in vitro*.

### HIF-1a increases brain cell survival via BNIP3 and NIX in OGDR *in vitro*

**Figure 8:**
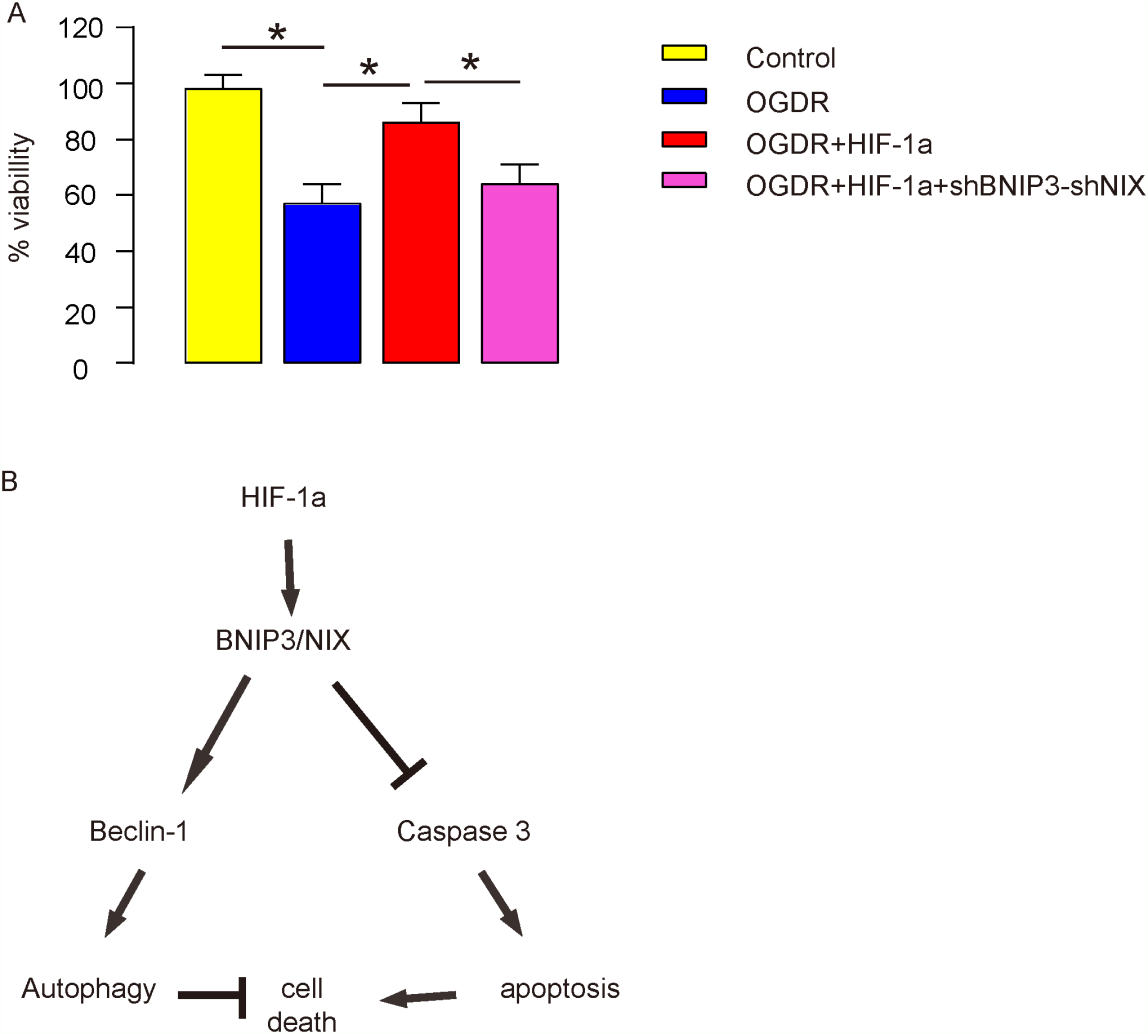
HIF-1a increases brain cell survival via BNIP3 and NIX in OGDR *in vitro* **(A)** Cell viability in a CCK-8 assay. **(B)** Schematic of the model: During IR, HIF-1a may have its protective effects through affecting brain cell apoptosis and cell autophagy, in a BNIP3 and NIX –dependent manner. ^*^p<0.05. N=5.

In order to find out the effects of HIF-1a on brain cell survival and the roles of BNIP3 and NIX in it, we examined cell viability in a CCK-8 assay. We found that HIF-1a significantly increased cell viability in OGDR, while depletion of BNIP3 and NIX significantly attenuated the protective effects of HIF-1a on cell viability (Figure 8A). Thus, these data suggest that during IR, HIF-1a may have its protective effects through affecting brain cell apoptosis and cell autophagy, in a BNIP3 and NIX –dependent manner (Figure 8B).

## DISCUSSION

HIF-1 is a heterodimeric protein composed of the HIF-1α and HIF-1β subunits [6–8]. The HIF transcriptional system has been shown as a key regulator of responses to oxygen level alteration and activates more than 70 genes that facilitate adaptation to ischemia and oxidative stress [6–8]. In addition, HIF-1 has also been proposed as a potential medicinal target for neurodegenerative diseases, including Alzheimer&s, Parkinson&s, and Huntington&s diseases as well as amyotrophic lateral sclerosis [6–8].

Although it is generally believed that ischemia induces HIF-1 expression, the role of HIF-1 in the ischemic brain is still controversial. HIF-1 and/or hypoxia have been reported to play either an anti-apoptotic or a pro-apoptotic role [6–8, 26, 27]. A very recent study showed that HIF-1 appeared to express in a two-phase manner after IR [28]. Early-phase HIF-1 expression resulted from increased protein stability, and it facilitated apoptosis. On the other hand, late-phase HIF-1 resulted from increases in gene transcription, and it promoted cell survival [28]. Moreover, the levels of HIF-1a that are induced in the experimental setting may be critical. For example, Dong et al. showed that HIF-1a had a protective effect against hippocampal apoptosis and cognitive dysfunction [26]. Hence, different mechanisms may control HIF-1 expression in the ischemic brain, and the outcome may be dependent on the exact experimental setting and the time points for analysis.

The previous studies had mainly focused on the study of brain cell apoptosis, while the effects of HIF-1a on autophagy remain unexplored. Indeed, recent studies have revealed the importance of cell autophagy in the protection of the brain cells [3, 15–21]. However, a role of HIF-1a in the regulation of brain cell autophagy after IR has not been studied. In the current study, we addressed this question.

We first used a well-established rat IR model, and we found that augmentation of HIF-1a significantly alleviated brain edema and improved in the outcome of neural function. Moreover, HIF-1a significantly increased two HIF-1a target genes BNIP3 and NIX, which appeared to be critical for HIF-1a-regulated cell apoptosis via caspases 3, and autophagic cell survival via Beclin-1. However, it may be difficult to evaluate the necessity of these two genes in the regulation of brain cell apoptosis and autophagy by HIF-1a *in vivo*. Hence, we used an *in vitro* model to mimic *in vivo* IR model. We chose a human cortical neuron cell line, which best mimicked the target cells in our *in vivo* model. HIF-1a activation *in vitro* was realized through plasmid-mediated HIF-1a expression in cells. Depletion of both BNIP3 and NIX were realized through shRNA-mediated gene knock-down. Of note, we have altered BNIP3 and NIX separately and found that the knockdown of either gene had lower effects, while knockdown of both nearly recapitulated the effects of depletion of HIF-1a. These data were not shown in the manuscript. Thus, BNIP3 and NIX may work in a redundant way downstream HIF-1a but a loss of both may defect the HIF-1a signaling in brain cells after IR.

We also found that both *in vitro* and *in vivo*, HIF-1a seemed to not only suppress brain cell apoptosis, but also increase cell autophagy. Thus, the effects of HIF-1a on brain cell survival appeared to be positive. Since apoptosis and autophagy are biological processes that are often regulated by similar pathways, it is expected that HIF-1a/BNIP3/NIX may inhibit cell death by favoring autophagy versus apoptosis. Thus, when HIF-1a is enhanced, the dominance of cell apoptosis vs autophagy is shifted.

Autophagy may have pro-survival and/or anti-survival roles, depending on interactions between apoptotic pathway and endoplasmic reticulum stresses, as well as the duration and strength of autophagy. This dual nature of autophagy has been discovered during analysis on the catabolic pathway in mammalian cells [29]. In line with this notion, the activation of the autophagic machinery may result in the death of cells. Hence, autophagic cell death is also termed as type-II programmed cell death, which is distinct from apoptosis as type-I programmed cell death [30]. However, the relationship between autophagy and apoptosis is very complex, since autophagy is not only able to collaborate with apoptosis to direct cell death, but also able to act as a survival mechanism. Very recent evidence suggests that prolonged autophagy in the absence of the central core of the apoptotic machinery could act as a cell survival mechanism to delay cell death in hematopoietic cells [31], while embryonic fibroblasts may utilize the autophagic machinery to undergo cell death [32], depending on the presence of apoptotic machinery. Hence, although starvation-induced autophagy is a mechanism tightly controlled, it appears that greater complexity of the signaling pathway interactions are involved in the regulation of cell death. Here, in our *in vitro* model, the effects on brain cell viability suggest that autophagy may favor cell survival in the current experimental setting for IR.

To summarize, our data suggest that augmentation of HIF-1a may ameliorate brain damages after IR through BNIP3 and NIX -dependent enhancement of autophagic cell survival, and may have a beneficial effect on the brain function preservation after IR.

## MATERIALS AND METHODS

### Protocol approval

The study protocol and experimental design was approved by the Ethical Committee for Animal Studies at Shanxi Provincial People&s Hospital. All rat experiments were approved by the Institutional Animal Care and Use Committee at Shanxi Provincial People&s Hospital (Animal Welfare Assurance). Surgeries were performed in accordance with the Principles of Laboratory Care Use (National Institute of Health), supervised by a qualified veterinarian.

### Rat treatment

Male Sprague–Dawley Rats (280–320g) obtained from the SLAC Laboratory Animal Co. Ltd (Shanghai, China) were fed on standard pellet chow and water. Animals were kept at 25°C, 50–60% humidity and a 12 h light/dark cycle prior to the experiments. Only water was given to the rats 12 h prior to experiments.

For IR model, rats were starved for 12 h prior to the surgical procedure. After anesthetization, carotid arteries of the rats were explored by performing merely neck incisions. For sham-operated group, the incisions were left open for 30 min and then closed. For IR group, bilateral femoral veins and arteries were opened by a cannula (no: 24; Datex/Ohmeda S/5, Helsinki-Finland) through inguinal incision. At day 1, both vertebral arteries were electrocauterized at the level of the first cervical vertebra within the alar foramina. After the closure of incision, the rats were wakened. At the second day (24 h later), the rats were anesthetized, after which CCA were bilaterally occluded with aneurysm clips for 30 min followed by 30 min of reperfusion. Rat brains were sampled after these treatments.

The rats were randomly assigned into 3 groups: sham group (n = 20), subjected to the neck incisions which were left open for 30 min and then closed; IR-HC group (n = 20), rats were infused with 10^7^ donor null-transduced hematopoietic cells (HC) 3 days before IR, and then subjected to IR procedure; IR+HC-HIF-1a group (n = 20), rats were infused with 10^7^ donor HIF-1a-transduced HCs 3 days before IR, and then subjected to IR procedure.

### Isolation, transduction and infusion of isogeneic HC to rats

Rat HCs were isolated from isogeneic rats, as described [33]. The isolated HCs were transduced with lentivirus carrying either null (as a control) or recombinant HIF-1a under a CMV promoter (Origene, Shanghai, China). For HC reinfusion, 10^7^ donor null/HIF-1a-transduced HCs were given to the receipt rats via tail vein 3 days before IR.

### Culture and treatment of human cortical neuron cells

HCN-1A is a human cortical neuron cell line, and was purchased from ATCC (ATCC, Rockville, MD, USA), and were cultured in Dulbecco's Modified Eagle's Medium (DMEM) supplemented with 20% fetal bovine serum (Invitrogen, Carlsbad, CA, USA) in a humidified chamber with 5% CO_2_ at 37°C. For generation of ischemic-like conditions by oxygen-glucose deprivation and reperfusion (OGDR) *in vitro*, cultures were placed in a hypoxia chamber containing an atmosphere of less than 0.2% O_2_, 5% CO_2_, 95% N_2_, >90% humidity, at 37°C. Within the chamber, the medium was removed and replaced with deoxygenated glucose-free Hanks’ Balanced Salt Solution (Invitrogen) for 2 hours, after which the cells were returned to normal culture conditions for 24 hours before analysis. Cells that were not exposed to OGDR condition were used as controls.

For induction of HIF-1a in HCN-1A cells, HIF-1a was expressed under a CMV promoter in a plasmid to be used to transfect the cells. Constructs for HIF-1a and short hairpin small interfering RNA for BNIP3 and NIX (shBNIP3 and shNIX) were all obtained from Origene. A plasmid carrying a scrambled sequence (scr) was used as a control for transfection. These constructs were generated and cloned into the TOPO plasmid (Invitrogen). The plasmids were transfected into cells at a concentration of 50 nmol/l using Lipofectamine-2000 (Invitrogen), receiving a nearly 100% transfection efficiency.

### Apoptosis assay by flow cytometry

For analysis of cell apoptosis, the cultured cells were re-suspended at a density of 10^6^ cells/ml in PBS. After double staining with FITC-Annexin V and propidium iodide (PI) from a FITC Annexin V Apoptosis Detection Kit I (Becton-Dickinson Biosciences, San Jose, CA, USA), cells were analyzed using FACScan flow cytometer (Becton-Dickinson Biosciences) equipped with Cell Quest software (Becton-Dickinson Biosciences) for determination of Annexin V+ PI- apoptotic cells.

### HPS

Routine paraffin embedding and sectioning (into 5μm-thick slice) of the dissected rat brains were performed. Hematoxylin and eosin (H&E) staining was performed, after which the histopathological scoring (HPS) of the hippocampus was carried out by a pathologist who is unaware of the groups as follows: grade 0, no damage to any hippocampal subregion; grade 1, scattered ischemic neurons in the CA1 subregion; grade 2, moderate ischemic damage; grade 3, whole pyramidal cell damage in the CA1 subregion; and grade 4, extensive cell damage in all hippocampal regions.

### Neurological scores

The neurological functions were evaluated on the basis of Modified Garcia Scale that has been previously described [34]. The mean of neurologic score was evaluated by two blinded observers independently.

### Brain water content

Rats were sacrificed under deep anesthesia 1% sodium pentobarbital (60 mg/kg, i.p.) at analysis. The brains were rapidly removed and weighed immediately (wet weight). Brains were then dried in an oven at 105°C for 24 h and weighed again (dry weight). Brain water content was calculated as [(wet weight - dry weight)/wet weight] X 100%.

### Determination of BBB permeability

BBB permeability was quantitatively evaluated by Evans blue (EB) extravasation at analysis. Briefly, rats were injected intravenously with 2% EB dye (5ml/kg, Sigma-Aldrich). One hour later, rats were anesthetized with sodium pentobarbital (60 mg/kg, i.p.) and perfused with PBS to remove the intravascular EB dye. The brain was then weighed, homogenized in PBS and centrifuged (15,000g, 30 min, 4°C). The supernatant was added to an equal volume of trichloroacetic acid with ethanol (1:3). The samples were incubated overnight at 4°C and then centrifuged (15,000g, 30 min, 4°C). The supernatant was quantified for absorbance of EB dye using a spectrophotometer (excitation 620 nm, emission 680 nm).

### Histology and TUNEL staining assay

Rats were deeply anesthetized and sacrificed by intracardial perfusion with PBS and 4% ice-cold paraformaldehyde (pH 7.4). The brain was quickly removed, immersed in 30% sucrose solution for at least 48 h, and then cut into 6μm thick coronal sections. Apoptotic cell death was detected using a TUNEL staining Kit (Roche Applied Science, Nutley, NJ, USA). Briefly, brain sections were incubated with 3% hydrogen peroxide (10 min), permeabilization solution (2 min), and TUNEL reaction mixture (60 min) at 37°C. Cell nuclei were stained with DAPI (1lg/ml, Roche Applied Science). Cell counting was performed in the ipsilateral basal cortex under a fluorescent microscope (Olympus). The total number of cells (DAPI+) and the TUNEL-positive cells were counted in 10 separate fields in 5 different slices. The data were expressed as the number of TUNEL-positive neurons per total number of neurons.

### Cell counting kit-8 (CCK-8) assay

The CCK-8 detection kit (Sigma-Aldrich) was used to measure cell viability according to the manufacturer's instructions. Briefly, cells were seeded in a 96-well microplate at a density of 5000/ml. After 24h, cells were treated with resveratrol. Subsequently, CCK-8 solution (20 ml/well) was added and the plate was incubated at 37°C for 2 h. The viable cells were counted by absorbance measurements with a monochromator microplate reader at a wavelength of 450 nm. The optical density value was reported as the percentage of cell viability in relation to the control group (set as 100%).

### Western blot and ELISA

Protein was extracted from the rat brain or the cultured cells with RIPA lysis buffer (Sigma-Aldrich) on ice. The supernatants were collected after centrifugation at 12000×g at 4°C for 20min. Protein concentration was determined using a BCA protein assay kit (Bio-rad, China), and the proteins were separated on SDS-polyacrylamide gels, and then transferred to a PVDF membrane. The membrane blots were first probed with a primary antibody. After incubation with horseradish peroxidase-conjugated second antibody, autoradiograms were prepared using the enhanced chemiluminescent system to visualize the protein antigen. The signals were recorded using X-ray film. Primary antibodies were rabbit anti-caspase 3, anti-Beclin-1, anti-LC3, anti-BNIP3, anti-NIX and anti-β-actin (Cell Signaling, San Jose, CA, USA). Secondary antibody is HRP-conjugated anti-rabbit (Jackson ImmunoResearch Labs, West Grove, PA, USA). β-actin was used as a protein loading control. The protein levels were first normalized to β-actin, and then normalized to the experimental controls. HIF-1a levels were determined by an ELISA kit (R&D System, Los Angeles, CA, USA).

### Statistical analysis

All of the statistical analyses were performed using the GraphPad Prism 6 (GraphPad Software, San Diego, CA, USA). Statistical analysis of group differences was carried out using a one-way analysis of variance (ANOVA) test followed by followed by Turkey multiple comparison post-hoc analysis. All values represent the mean ± standard deviation (SD). A value of p<0.05 was considered statistically significant after Bonferroni correction.
